# RANSAC for Robotic Applications: A Survey

**DOI:** 10.3390/s23010327

**Published:** 2022-12-28

**Authors:** José María Martínez-Otzeta, Itsaso Rodríguez-Moreno, Iñigo Mendialdua, Basilio Sierra

**Affiliations:** 1 Department of Computer Science and Artificial Intelligence, University of the Basque Country, 20018 Donostia-San Sebastián, Spain; 2 Department of Languages and Information Systems, University of the Basque Country, 20018 Donostia-San Sebastián, Spain

**Keywords:** RANSAC, feature matching, transformation matrix, shape detection, object recognition, robotic systems, real time

## Abstract

Random Sample Consensus, most commonly abbreviated as RANSAC, is a robust estimation method for the parameters of a model contaminated by a sizable percentage of outliers. In its simplest form, the process starts with a sampling of the minimum data needed to perform an estimation, followed by an evaluation of its adequacy, and further repetitions of this process until some stopping criterion is met. Multiple variants have been proposed in which this workflow is modified, typically tweaking one or several of these steps for improvements in computing time or the quality of the estimation of the parameters. RANSAC is widely applied in the field of robotics, for example, for finding geometric shapes (planes, cylinders, spheres, etc.) in cloud points or for estimating the best transformation between different camera views. In this paper, we present a review of the current state of the art of RANSAC family methods with a special interest in applications in robotics.

## 1. Introduction

The Random Sample Consensus algorithm, commonly known by its acronym RANSAC, was developed by Fischler and Bolles more than forty years ago as a novel approach to the robust estimation of the parameters of a model in regression analysis [1]. It addresses situations where there is a high percentage of outliers in the data, which hinders the parameter estimation task. While other approaches, such as least squares linear regression, use all the available data to produce a model which could be later refined, RANSAC creates several models in sequence, each time choosing, from the available data, a random sample of the minimum size needed to create a model. After each step in the sequence, the support of the model is calculated, typically splitting the data into inliers or outliers, the former are data points whose measure of fitness with respect to the model fall below a certain threshold, and the latter are those which do not comply with that requirement. For example, if the task is to estimate the parameters of a plane in a point cloud, RANSAC would sample three points, the minimum needed to define a plane (provided they are not all collinear with each other), then would compute the euclidean distance of all the points in the point cloud to such a plane, and then the percentage of inliers according to some threshold. After some number of iterations, RANSAC would return the plane with the highest support, thus the plane with more points close to it.

Several questions and practical issues arise from this simple definition. It is not obvious at all how to choose a reasonable threshold to discriminate between inliers and outliers, or how to determine the number of iterations through the process of creating and evaluating a model. The computational cost could also skyrocket if the whole dataset has to be checked against the hypothesis model at each iteration. For these reasons, researchers have devised a number of variations over this vanilla RANSAC trying to address these issues.

The range of problems for which RANSAC is well-suited is very wide, although in robotic applications they usually pertain to two big classes: shape detection and feature matching. Geometric models of simple shapes such as planes, spheres, cylinders, etc., are well understood and relatively straightforward to implement, while of relevant practical interest, due to the fact that human-made objects and some natural ones are close to those shapes. Therefore, the ability to detect these geometric structures in 3D data is of great importance for environment understanding in indoor or outdoor robotic navigation and/or mapping. A high-performant feature-matching procedure is also very desirable for finding the right transformation between different views of a scene. Features are extracted from several scenes taken from different points of view, and the task is defined as finding the matrix transformation between images that minimizes the distance between matching features.

Other machine learning approaches, such as deep neural networks [2,3], while extremely successful for object detection, can be difficult to fine-tune or interpret. Their main advantage is that they could be readily applied to problems in which there is no restriction on the object shapes, but a RANSAC implementation can be more efficient when the shape is simple enough to be expressed as a model with a few parameters. As a result, the fields of application of these two kinds of methods are usually different, although it is possible to apply RANSAC as a preprocessing step before applying deep learning techniques. For example, a deep learning application for point cloud segmentation could benefit from RANSAC filtering the floor or the walls of the scene.

Several studies have been carried out on the performance of RANSAC methods. In [4], the authors classify the RANSAC variants into three types, depending on the intended improvement over the vanilla version: accuracy of the model, computational speed, and robustness with respect to the choice of the number of iterations and the threshold value. They analyze the performance of ten variants on a line fitting synthetic data and on a 2D homography estimation on real data, comparing also with Least Median of Squares [5] and projection-based M-estimator [6]. Their results show the existence of a trade-off of accuracy and robustness over computing time. In [7], the authors present a comparison of eleven variants on the problem of finding planes in 3D point clouds. Their findings suggest a trade-off similar to those noticed in [4].

Our goal is to update the list of RANSAC variants with more recent development and, at the same time, present practical applications that could be of interest to the robotics practitioner. The reader is also informed of existing open-source software that could be of interest. Due to the plethora of RANSAC variants developed by the community, it is impossible to list them all. We tried our best to present the most used, cited, or influential ones in further developments. Some algorithms that might deserve a mention, such as RAMOSAC [8] or KALMANSAC [9], were left out, as they were specifically designed for one application (target tracking in those mentioned above) and lack the generality of applications of those presented in Section 3.

In this paper, we present a survey of RANSAC-like methods with a focus on shape detection and image matching for robotic applications. First, we describe the vanilla RANSAC algorithm in detail, along with several variations that have been devised to try to address some of its limitations. Then, we review some recent applications, also pointing out some open-source software that could be of interest to the researchers interested in this field. Finally, we discuss the current state of the art and present our conclusions.

## 2. RANSAC

Fischler and Bolles’s Random Sample Consensus (RANSAC) algorithm is a general parameter estimation method designed to handle data where a high percentage of outliers is present [1]. RANSAC was created by members of the computer vision community [10], in contrast to many other popular robust estimating methods also embraced by that community, as in the case of M-estimators [11] and Least Median of Squares [12].

It is a resampling method that produces potential solutions by using the fewest possible observations to estimate the model’s underlying parameters. RANSAC employs the smallest possible set of observations and then expands this set with consistent data points, in contrast to typical sampling strategies that use all the available data to generate an initial solution proposal and then refine the model deleting outliers.

The vanilla RANSAC pseudocode is described in Algorithm 1. The condition that must meet a point to be an inlier with respect to the model being evaluated is that the point “fits well”. As the most common applications of RANSAC are related to finding shapes or matching features between different views, it is usual to refer to the euclidean distance from a point to the model. However, any other function that measures the concordance between the model and the point might be used. As it can be observed in Algorithm 1, the time complexity of the vanilla RANSAC algorithm is linear in the product of the number of iterations by the number of data points, and therefore it could be computationally expensive.
**Algorithm 1:** RANSAC algorithm
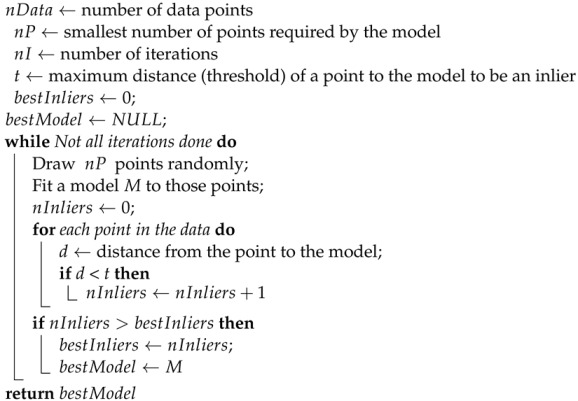


In order to tackle this problem, a very common modification of this algorithm consists of defining a measure of goodness of a model such that, if in any of the iterations a model good enough is found, the procedure terminates and returns that model as the solution. For example, it would be possible to compute the percentage of inliers over the total number of data points and decide that if a model exceeds some predefined threshold, the model is suitable for our goal and the search ends.

When it is not clear what could be a criterion for considering a model good enough, or when all the models are of low quality, but we are interested in the best among them, the natural question that arises is how to choose the number of iterations. Let us denote the inlier ratio over the total data points as iRatio. The probability that in a set of nP randomly chosen points all are inliers is of $iRatio^{nP}$, and therefore the probability of this situation not happening in an iteration is of $1-iRatio^{nP}$, and in nI iterations of ${\left(1-iRatio^{nP}\right)}^{nI}$. As we want this probability to be small, nI has to be chosen such that ${\left(1-iRatio^{nP}\right)}^{nI}<\alpha$, where α might be 0.05 for a probability of 95% of randomly drawing all inlier points in some iteration. After some algebraic manipulation, we find the following equation:

$$nI=\frac{log(\alpha )}{log(1-iRatio^{nP})}$$

Unfortunately, the ratio of inlier points is often not known in advance and the usefulness of the previous result is limited. In Section 3.2, several approaches focused on improving the speed of the algorithm are described, empirically showing that it is often possible to run RANSAC well below its theoretical time complexity. Some authors even claim constant time model evaluation [13].

### 2.1. Matching Images

One common problem in computer vision is finding the right correspondences between features of two images of the same scene taken by different cameras. Epipolar geometry is the branch of geometry that helps to formalize the relationship between cameras, points in the 3D space, and their projections in the camera views.

**Figure 1:**
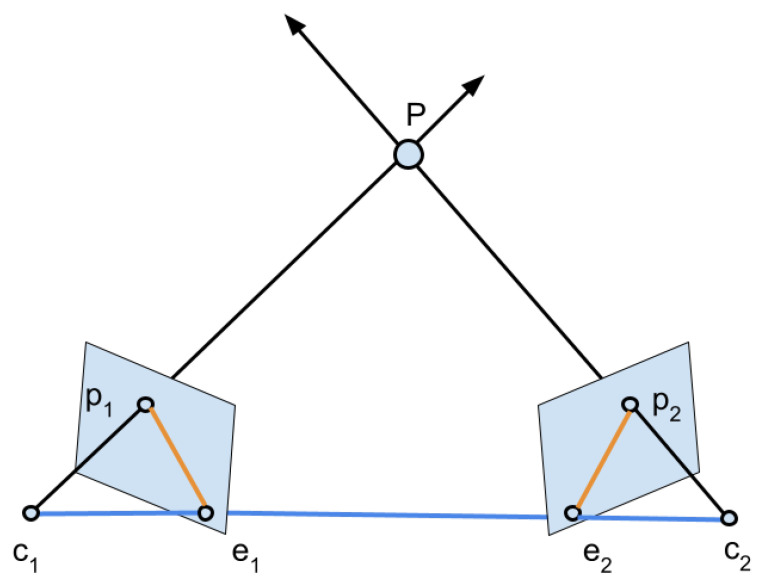
The general setup of epipolar geometry. The planar region defined by the points *P*, $c_{1}$, and $c_{2}$ is the epipolar plane. The blue line is the baseline, while the two orange lines are the epipolar lines.

In Figure 1, a typical situation is shown where two cameras (or the same camera at different timestamps) observe a point P located at some distance in the space. The camera centers are $c_{1}$ and $c_{2}$, where the projections of the point P in the image planes are $p_{1}$ and $p_{2}$, respectively. The line connecting the two camera centers is referred to as the baseline, while the plane defined by $c_{1}$, $c_{2}$, and *P* is the epipolar plane. The intersection points between the baseline and the two image planes are denoted as the epipoles $e_{1}$ and $e_{2}$. The epipolar lines are defined by the intersection of the epipolar plane with the image planes, and they intersect the baseline at the epipoles. When the two image planes are parallel, then the epipoles $e_{1}$ and $e_{2}$ are located at infinity.

The transformation from one view to the other is given by a homography matrix that is necessary to estimate. To do so, typically, some algorithm for finding features, such as SIFT [14], is employed, and then a model fitting procedure is conducted.

### 2.2. Finding 2D/3D Shapes

**Figure 2:**
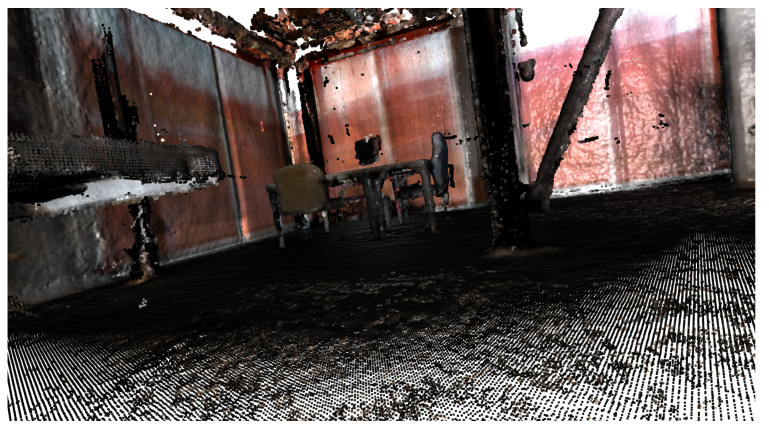
Visualization of a point cloud of a room.

Basic geometric shapes are the constituent parts of multiple objects in the environments in which robots usually perform their tasks. Walls, floors, and ceilings are examples of planes located in indoor settings. Doors, windows, tables, and stairs are also composed of planes, while pipes could be an example of cylinders. In urban outdoors, roads, buildings, or traffic lights also resemble geometric shapes. Even in agricultural areas, the crops use to be organized in lines or trees could be modeled as conical structures. In Figure 2, several objects in a room can be seen, including the walls and columns. There is also noise in the point cloud, a typical circumstance that could be addressed using RANSAC to detect shapes.

## 3. RANSAC Variants

It is difficult to classify the plethora of RANSAC variants for several reasons. First of all, sometimes, a previously known variant is employed as part of a pipeline with a small tweak dependent on the specific problem or the rest of the pipeline. Methods that clearly fall into this category are described in the section corresponding to applications. Secondly, some methods try to improve the vanilla RANSAC in more than one dimension, for example, achieving better accuracy while at the same time yielding a lower computational load and being faster. This paper does not intend to provide an exhaustive catalog of all the existing variants but to present a state-of-the-art survey of the most commonly used, along with some applications in the robotic field.

Following the classifications previously made in [4,7], we divide the methods into four types, depending on which is the main area they intend to improve: accuracy, speed, robustness, and optimality. As mentioned above, sometimes the distinction is not clear-cut, but when possible, we followed the previous surveys. When the authors have developed a method that is specifically designed for a concrete application, and not as a general estimation method, we present it in the Applications section. A summary of the variants grouped by areas is presented in Table 1.

### 3.1. Accuracy-Focused Variants

#### 3.1.1. MSAC (M-Estimator SAC)

MSAC [15] is the first iteration of a family of RANSAC variants that uses maximum likelihood estimates for the parameter models. The authors tackle the problem of the estimation of the trifocal tensor which, given correspondences between points in two images, determines the position of such points in a third image. Their results show an increase in accuracy with the downside of degradation of computational performance. They claim their method could be used for any other problem in computer vision, mentioning the fundamental matrix estimation task as an example.

#### 3.1.2. MLESAC (Maximum Likelihood SAC)

The RANSAC algorithm looks for the model that maximizes the number of inliers. MLESAC [16] computes the log-likelihood of the model, taking into account the distribution of outliers, and uses random sampling to maximize it. The authors show the usefulness of their approach by deriving the log-likelihood for the problem of estimating the fundamental matrix in a two-view problem and implementing the algorithm, obtaining good results.

#### 3.1.3. MAPSAC (Maximum A Posterior Estimation SAC)

A Bayesian approach is presented in [17], from the same authors of MSAC and MLESAC, with the aim of improving over their previous maximum likelihood formulations. They develop MAPSAC to obtain a robust Maximum A Posterior (MAP) estimate of the problem of the least square fitting of an arbitrary manifold, and in particular, of lines or planes. A new method for approximating the posterior probability of a model, called GRIC, is derived, and it is theoretically and empirically demonstrated that it is more accurate than AIC [38], BIC [39], and MDL [40] in tasks where a large number of latent variables is present.

#### 3.1.4. LO-RANSAC (Locally Optimized RANSAC)

In [18], the authors introduce Locally Optimized RANSAC (LO-RANSAC) to address the empirical observation that the number of samples needed to find an optimal solution with a given probability is significantly higher than the amount predicted by the theory [41]. They realize that a commonly held assumption is incorrect: that a model computed from a sample composed only of inliers has to be consistent with the whole set of inliers.

In spite of the computed model not being optimal, in practice, it is sufficiently close to the optimal model for a local optimization method to approach or even find it. After the optimization step, the model covers a greater amount of inliers, and therefore reduces the number of steps of the RANSAC method, making it approach to its theoretical value. The optimization strategy guarantees to keep the number of used samples very low, hence, accordingly, the extra time spent in each step is almost negligible.

Their proposed optimization algorithm consists of the following steps:

1. Define a threshold θ and a number of optimization iterations *I*.
2. In each step of the RANSAC method, the samples are selected only from the data points that are consistent with the model created in the previous step.
3. Take all data points with error smaller than I×θ and compute new model parameters according to a linear algorithm. Reduce *I* by one and iterate until the threshold is θ.

This local optimization step is only performed when the number of inliers in the current RANSAC step is greater than the previous maximum. The number of points from a randomly drawn sample that are consistent with a given model is a random variable with usually unknown probability density function. As this density function is the same for all samples with the same discrete cardinality, the probability that the *k*th sample will be the best among the already drawn samples is $\frac{1}{k}$. Therefore, the average number of samples which are the best so far is a sequence of *n* samples:

$$\sum_{1}^{n}\frac{1}{n}\leq \int_{1}^{n}\frac{1}{n}dx+1=\log\;n+1$$

The authors perform experiments on epipolar geometry and homography estimation and find that the empirical results are close to this theoretical average, while speeding up the RANSAC procedure by two to three times. The number of inliers of the solution found, which can be thought as a proxy for its overall quality, is increased in the range of 10–20%.

#### 3.1.5. QDEGSAC (RANSAC for Quasi-Degenerate Data)

Sometimes, when confronted with the problem of computing the fundamental matrix for image matching coming from different views, the data do not provide enough constraints to find a unique solution, but only up to a set of solutions that all of them could explain the data. To tackle this problem, in [19], QDEGSAC is presented to cope with the problem of degenerate data. The authors develop a hierarchical RANSAC over the number of present constraints that do not require problem-dependant tests. They claim results similar to other approaches that leverage knowledge about the degeneracy source.

#### 3.1.6. Graph-Cut RANSAC

A modification of LO-RANSAC is presented in [20], where the graph-cut algorithm [42] is applied in the local optimization step to the best model obtained till that moment. The motivation is to separate inliers and outliers. The authors test its adequacy to several computer vision problems with synthetic and real data and claim it to be more geometrically accurate and at the same time easy to implement. Additional improvements have been presented in [43,44], where USAC [45] and MAGSAC++ [46] robust estimators are included in the algorithm.

### 3.2. Speed-Focused Variants

#### 3.2.1. NAPSAC (N Adjacent Points SAmple Consensus)

The premise behind NAPSAC [21] is that inliers tend to be closer among them than with respect to the outliers. Therefore, instead of picking completely random samples to generate the models, a better strategy could take into account this fact. The authors suggest taking an initial point randomly and then finding the number of points lying within a hypersphere of radius *r* centered on that point. If the number of points in such a hypersphere is fewer than the minimal set needed to estimate the parameters of the model, then fail and choose another initial point; otherwise, select the initial point and other points uniformly from the set of points inside the hypersphere until the minimal number needed to estimate the model have been selected. The authors derive optimal values for the radius of the hypersphere if the inliers are perturbed by Gaussian noise and the outliers are distributed uniformly in the hypersphere.

#### 3.2.2. Randomized RANSAC with $T_{d,d}$ Test

The hypothesis test step in the RANSAC algorithm is often very costly. If a plane has to be fitted against a point cloud containing millions of points, the distance to every point has to be computed to assess the goodness of the hypothesized plane model. To deal with this issue, in [22], the authors propose an algorithm that only evaluates a fraction of the data points. They define a $T_{d,d}$ test that is passed if all the *d* randomly selected data points are consistent with the hypothesis currently being tested. The optimal value of *d* is computed with the following expression

$$d\approx \frac{\ln(\frac{\ln\epsilon (t_{M}+1)}{N(\ln\delta -\ln\epsilon )})}{\ln\delta },$$

where $t_{M}$ is the time necessary to compute the parameters of the model from a sample, δ is the probability that a data point is consistent with a random model, and ϵ is the fraction of outliers in the data. As *d* has to be an integer, $d_{opt}$ is chosen as the number in {⌊d⌋,⌈d⌉}, which minimizes the previous expression, provided it is greater than zero. One drawback of this approach is that an estimation of the fraction of outliers in the original data is needed.

#### 3.2.3. Guided-MLESAC

A limitation of the maximum-likelihood estimation in MLSAC is that it does not take into account possible knowledge about the prior probabilities of the parameters of the model to be estimated. In [23], the authors propose guided-MLESAC, where a good estimation of the prior probabilities is shown to give an order of magnitude speed improvement in the problem of finding correspondences between features in images taken from different views. After a theoretical analysis and experiments to compute the priors, their conclusions are that, with little extra computation, it is possible to leverage quality measures provided by image matcher software to derive confidence in the validity of a match and incorporate them as priors. This knowledge can be useful to select more probable hypotheses and also to compute more accurately the cost of fitting.

#### 3.2.4. RANSAC with Bail-Out Test

In [24], the author, inspired by the randomized RANSAC with $T_{d,d}$ test, describes a modification of the RANSAC procedure that allows the scoring process to be terminated earlier, and therefore could save computational time. He first defines a trivial early bail test, in which a hypothesis is not further checked against the remaining data if its current score cannot improve the score of the best hypothesis tested so far. Then, he proceeds to propose a test in which a randomly selected subset of size *n* is evaluated, and its fraction of inliers ($\epsilon_{n}$) is computed. If $\epsilon_{n}$ is clearly smaller than the best $\epsilon_{best}$ found so far, it is very unlikely that evaluating the rest of the points would produce a better result than $\epsilon_{best}$. In the paper, estimates for the probability of ϵ improving over $\epsilon_{best}$ when evaluating the remaining points are derived under the supposition that the number of inliers contained in a subset of size *n* follows a hypergeometric distribution. Experiments show a significant reduction in the number of evaluations with respect to the $T_{d,d}$ test approach.

#### 3.2.5. Randomized RANSAC with Sequential Probability Ratio Test

For randomized models to work properly, usually an estimate of the fraction of inliers in the data is needed. In [25], another approach that does not require such knowledge is presented. The authors base their work on Wald’s theory of sequential decision making [47], deriving a process to generate a solution with confidence 1−η, where η is a probability decided by the user. Wald’s Sequential Probability Test Ratio is based on the likelihood ratio

$$\lambda_{i}=\prod_{n=1}^{i}\frac{p(x_{n}|H_{b})}{p(x_{n}|H_{g})}=\lambda_{i-1}\frac{p(x_{i}|H_{b})}{p(x_{i}|H_{g})},$$

where $H_{g}$ is the hypothesis that the model is good, i.e., computed from a sample composed only of inliers, and $H_{b}$ corresponds to the alternative hypothesis that the model is considered bad. The variable $x_{n}$ is equal to 1 if the n-th data point is consistent with the evaluated model, and 0 otherwise. The probability $p(1|H_{g})$ that a random point is consistent with a good model is approximately the percentage of inliers in the original data, represented as ϵ, and the probability of being coherent with a bad model is modeled as a Bernouilli distribution with parameter δ = $p(1|H_{b})$. Given that the majority of all models tested by RANSAC are bad in the former sense, δ can be estimated as the average percentage of consistent data points in rejected models. On the other hand, a lower bound on ϵ is given by the size of the largest support for the considered models so far. Experiments show that this method is from 2.8 to 10 times faster than RANSAC and up to 4 times faster than the Randomized RANSAC with $T_{d,d}$ test.

#### 3.2.6. PROSAC (Progressive Sample Consensus)

PROSAC [26] establishes a rank of promising data points (and therefore, of promising hypotheses or models) according to some measure of the quality of the data. As the hypothesis testing procedure advances, the confidence in the adequacy of the quality scores decreases, and the sampling strategy is shifting toward the original RANSAC. The more promising samples are drawn at the earlier stages, but in further steps, data points with lower quality scores are gradually incorporated until all the original samples have a nonzero probability of being drawn. The authors employ PROSAC for the task of estimating the correspondences between features in two images from different camera views. They claim to achieve significant time savings over RANSAC, in the order of hundreds of times, due to the fact that good hypotheses are generated early on in the sampling process.

#### 3.2.7. GASAC (Genetic Algorithm SAC)

In [27], the authors propose an approach based on genetic algorithms, where a population of sets of parameters evolves to yield a solution. They tackle the problem of finding the fundamental matrix associated with different views of a scene, but the method can be adapted to any problem usually solved by RANSAC. The individuals of the genetic pool are characterized by a chromosome in which the model parameters are encoded and are subject to the usual crossover and mutation operators. A number of evaluations of an order of magnitude less than with the usual RANSAC are reported, when tested on several image-matching problems.

#### 3.2.8. One-Point RANSAC

While multiple works have analyzed the way to reduce the number of data points against which to test the hypothesized models, some researchers have worked on how to reduce the number of data points needed to generate a hypothesis. In [28], the authors propose a method to generate a hypothesis from just a data point by leveraging a priori information from an extended Kalman filter [48]. Experiments are performed in two scenarios: the first one is a six-degree-of-freedom motion estimation from a monocular sequence, and the second one is a robot trajectory estimation combining wheel odometry and monocular vision. The authors claim results comparable to other visual odometry methods.

#### 3.2.9. GCSAC (Geometrical Constraint SAmple Consensus)

Geometric constraints could be used to select good samples to generate models that would be further tested for consistency against the data. In [29], the authors present a method that searches for such samples following two criteria: the selected samples must be consistent with the estimated model according to an inlier ratio evaluation and, at the same time, they must satisfy geometrical constraints of the object we are looking for. Experiments were performed for cylinder fitting in several datasets, one of them synthetic, the second one consisting of data obtained in their laboratory, and the third one from public datasets. Their results demonstrate better accuracy than MLSAC and real-time performance.

#### 3.2.10. Latent RANSAC

An attempt to evaluate a RANSAC-generated model in constant time, independently of the size of the data set, is presented in [13]. The authors’ insight is that the correct hypotheses form clusters in the latent parameter domain. From this observation, an approach similar to the randomized version of the generalized Hough transform [49] can be applied to find those clusters, claiming that only two votes are necessary to succeed in the search. The fast localization of the pairs of similar hypotheses is possible thanks to an adaptation of the random grids search technique [50]. Therefore, the computationally demanding hypothesis verification stage only takes place after the discovery of a similar pair of them, and it is shown that this event is very rare when the hypotheses are incorrect. The authors perform experiments on three different types of problems on both synthetic and real data: camera localization, 3D rigid alignment, and 2D-homography estimation. They claim an improvement in speed without degradation in accuracy.

### 3.3. Robustness-Focused Variants

#### 3.3.1. AMLESAC

A noise-adaptive variant of MLESAC [16] is presented in [30]. It applies the sampling strategy of MLESAC, and also searches for the model that maximizes the likelihood. The improvement over it is the simultaneous estimation of the percentage of inliers (γ) and the standard deviation of the noise affecting the inliers (σ). This is achieved by defining as a function of γ and σ the log-likelihood of all points under a hypothesis $\theta_{k}$ and selecting the values that maximize it. Then, the likelihood of $\theta_{k}$ using all the data and the previously estimated values for γ and σ is computed. All the process is repeated *M* times, where *M* was obtained based on a prior estimate of γ, and the hypothesis θ with the highest likelihood among the $\theta_{k}$ obtained in each iteration is returned. Then, the model is refined by applying nonlinear minimization using point-based parameterization [51]. Experiments on synthetic and real data show that AMLESAC outperforms previous methods for the pose estimation task without relying on the knowledge of noise parameters.

#### 3.3.2. u-MLESAC

Another method based on MLESAC is u-MLESAC [31]. As in MLESAC, it can be decomposed into four steps: sampling of the data, estimation of the parameters, estimation of the variables of the error model, and evaluation of the parameters according to the maximum likelihood criterion. The novelties of u-MLESAC are the estimation both of the variance of the error model and of the number of iterations. The variance σ of the error model is estimated by the expectation–maximization (EM) algorithm [52], and the number of iterations is computed from the condition that all the sampled data are inliers and within a desired error tolerance β. The number *t* of iterations is computed as

$$t=\frac{\log\;\alpha }{\log\;(1-k^{m}\gamma^{m})},$$

where *m* is the number of sampled data points, γ is the inlier ratio, and $k=\mathrm{erf}(\frac{\beta }{\sqrt{(}2)\sigma })$, with erf as the Gauss error function. Experiments with line fitting tasks showed high accuracy and robustness in different data distributions.

#### 3.3.3. Recursive RANSAC

The standard RANSAC algorithm assumes that all the data are available at the start of the estimation process. To tackle the problem of data appearing sequentially, in [32], the Recursive RANSAC algorithm is presented. The authors point out that, as the recursive least-squares algorithm (RLS) [53] is the extension of the least-squares method to sequential data, Recursive RANSAC is the recursive version of RANSAC, with the added capability of being able to track multiple signals simultaneously. This approach makes use of RANSAC to estimate models that fit the current observations with previous observations. When an observation is an inlier to a model, the model is updated by means of recursive least squares. Experiments with simulated data show that Recursive RANSAC is more accurate than RLS, Hough transform [54], and batch RANSAC [55] when the task is to estimate the parameters of a single random line. Another simulation of multiple signals tracking in the task of geolocating stationary ground objects using aerial sensors shows promising results.

#### 3.3.4. SC-RANSAC (Spatial Consistency RANSAC)

The authors of SC-RANSAC [33] present a robust and efficient method to detect points that are clearly outliers, with the aim of removing them and therefore increasing the inlier ratio in the data given to RANSAC. To detect those outliers, the method takes advantage of spatial relations between corresponding points in two images. This approach can also be seen as a preprocessing step for other RANSAC variants. Experiments performed over standard datasets of real images show improvements in computational time and also in accuracy over other methods such as RANSAC and PROSAC. These advantages are especially noticeable when the percentage of outliers is high.

#### 3.3.5. NG-RANSAC (Neural-Guided RANSAC)

The field of neural networks permeates every area of machine learning and parameter estimation nowadays, and RANSAC research is not an exception. NG-RANSAC [34] is a RANSAC variant that uses prior information to guide the search of model hypotheses, with the aim of increasing the probability of finding sets with no outliers or very few of them. In other approaches, the prior information is obtained by heuristic methods that make use of hand-designed descriptors, built from the domain knowledge of the researcher. In contrast, NG-RANSAC uses neural networks to navigate through the set of hypotheses. Self-supervision of the process is achieved using the inlier percentage as part of the training data, and the addition of a differentiable version of RANSAC allows for further improvements. Experiments on fundamental matrix estimation, camera relocalization, and horizon line estimation achieve state-of-the-art results.

#### 3.3.6. LP-RANSAC (Locality-Preserving RANSAC)

In [35], the authors integrate a locality-preserving constraint into the RANSAC workflow, with the goal of pruning unreliable hypotheses before the scoring loop and also guiding nonuniform sampling to generate and score more promising models earlier. Experiments on public datasets yield more accurate and stable solutions than other state-of-the-art methods, this advantage being more evident when there is a low inlier percentage. The locality-preserving constraint is derived from the work in [56], which observed that in two images of the same scene taken under different points of view, the absolute distance between two feature points may change greatly, but their relative location is much better preserved due to physical constraints. The guided sampling strategy makes use of the locality-preserving scores to assign more weight to more promising areas in the search space.

### 3.4. Optimality-Focused Variants

#### 3.4.1. Optimal Randomized RANSAC

In [36], the authors present a randomized version of RANSAC and prove that it is optimal regarding a probability estimated by the user. The time spent to arrive at a solution is close to the minimum possible and better than any deterministic strategy. In fact, the algorithm is the fastest possible, in the average case, among all randomized algorithms when the proportion of inliers is known in advance. The algorithm is a version of Randomized RANSAC with SPRT test [25], the improvement being that the optimal decision threshold for deciding if a model is good or bad is also derived and not left to the user as a parameter to set up.

#### 3.4.2. Optimal RANSAC

Another algorithm that finds the optimal model in nearly every run in some kinds of problems is presented in [37]. The authors present an approach with some similarities to LO-RANSAC [18], as both of them conduct repeated resampling on the set of tentative inliers performing iterative estimation of the model. The differences are: the optimization is performed only when the tentative set has more than five inliers, in order to avoid little promissory sets when there is a low inlier ratio; when a larger set is found when resampling, the resampling starts again with that set, so the set will grow until the largest set is found thanks to iterative re-estimation and rescoring; the iteration process continues until the set no longer changes, which yields a high probability that the found set is optimal; a pruning step is finally performed with a low tolerance, in order to preserve only the best inliers; the model is recomputed from the remaining inliers in each iteration. Experiments with line finding in aerial images show optimal solutions in more than 99.95% of the cases.

## 4. Applications

RANSAC or any of its variants have been used in many different kinds of applications as a solution to implement shape detectors or to find the correspondence between features extracted from images taken from different points of view. In this section, we present some of the research described in the literature. We organized the applications into three groups: image matching, shape detection, and hardware acceleration. The first two groups correspond to the two main areas in which RANSAC has been applied in robotic applications, while in the third one, we present all the implementations that make use of parallelism or special hardware. As far as the hardware acceleration does not rely on a specific kind of application, we deemed it appropriate to group them in a dedicated section.

### 4.1. Image Matching

Finding the correspondence between feature points in two images is a problem of great interest in the field of robotics, with applications in indoor as well as outdoor environments. Simultaneous Localization and Mapping (SLAM) is the family of techniques that allow a mobile robot to build a map of the environment and localize inside it at the same time [57]. To deal with SLAM in dynamic environments, in [58], the authors present a variant of RANSAC, called multilevel-RANSAC (ML-RANSAC), to classify objects into static or dynamic. The main advantage of their approach is that it can address both static and dynamic objects in SLAM and detect and track moving objects without the need of splitting the problem. The ML-RANSAC method takes as input in time step *n* the estimated state and covariance at step n−1 according to an Extended Kalman Filter, the sensors measurements at time step *n*, a threshold value to decide if a detected object is a track of a previous one, and the maximum number of desired iterations. It is also needed to provide the kinematic models of the robot, stationary and dynamic objects, as well as the observation model. ML-RANSAC outputs the estimated state and the number of static and moving objects, as well as the covariance of all these entities at the time *n*. Experiments performed in simulation and with a Pioneer P3-DX robot in an indoor dynamic setting show that this approach can reliably estimate the robot’s pose while building the map and keeping track of moving objects. In [59], the authors present further developments over their previous work, adding a layer of object detection and classification using machine learning, more precisely convolutional neural networks (CNN). A CNN is trained to detect doors and people, and the pipeline is tested in a real environment, yielding promising results.

The problem of removing erroneous or redundant matches in SLAM has also been tackled with RANSAC. In [60]; the authors present GMS-RANSAC, an algorithm to remove the mismatches based on oriented fast and rotated brief (ORB) in SLAM [61]. The key idea behind the grid-based motion statistics (GMS) algorithm [62] is the realization that adjacent pixels in images taken from different points of view share a similar motion, and that those relationships can be defined as smoothing constraints and be combined into a statistical framework to reject erroneous matching. Therefore, good correspondences are associated with a high number of similar neighbors in a 3D region. The main problem with this approach is that when there are few points in each 3D grid, the confidence is low and the number of errors could increase. The addition of RANSAC to the method allows for more robust results when the dataset is challenging to GMS. Experiments on public datasets show an average correction rate of 28.81% over the GMS algorithm.

Another problem of interest for robotic applications such as camera calibration, scene tracking, or robot navigation is the detection of vanishing points in images [63]. Due to perspective, lines that are parallel in 3D space appear to converge to a point called a vanishing point when projected in a 2D space. In [64], the authors propose a new RANSAC variant, called under-parameterized RANSAC (UPRANSAC) which, combined with the Hough transform, is able to detect vanishing points in uncalibrated monocular images in real time. The degrees of freedom of a vanishing point are found first by applying UPRANSAC to choose a hypothetical inlier and compute a portion of the degrees of freedom and then executing the voting scheme associated with a 1D Hough transform to find the remaining degrees of freedom along the extension line of the previously hypothesized inlier. Vanilla RANSAC selects two edges as a hypothetical pair of inliers and needs both of them to be right to fit a correct model of vanishing points, while UPRANSAC has a higher likelihood of finding one inlier and, therefore, is more reliable in this task. Experiments on public datasets show high accuracy and real-time performance.

Dense alignment between two images is the goal of the work described in [65]. The authors start from the observation that parametric and nonparametric alignment methods have different strengths that are complementary to each other. Then, they propose a two-stage method, where a feature-based parametric coarse alignment is followed by a nonparametric fine alignment. The coarse alignment is performed by RANSAC estimating the transformation matrix from deep features, and the fine alignment is learned at the pixel level in an unsupervised way by a deep neural network that tries to optimize a standard structural similarity metric between the two given images. The deep features for the coarse alignment stage are the conv4 layer of a ResNet-50 network, while in the fine alignment stage, the goal is to find a flow that warps the image source into an image similar to the target, with that similarity being measured by structural similarity [66]. The authors claim good results on a range of tasks, including unsupervised optical flow on KITTI [67], dense correspondence on HPatches [68], and two-view geometry estimation on YFCC100M [69], among others.

A parallel robot, also called parallel manipulator, or generalized Stewart platform, is a mechatronics device that supports a single platform using several serial chains controlled by a computer system [70]. The parallel aspect of the robot is not related to its geometric appearance but to the fact that several actuators could work in parallel, affecting the platform at the same time. In [71], the authors present a system that employs Harris-SIFT [72] and RANSAC to detect the pose of a parallel robot with three degrees of freedom which was developed by them. Harris-SIFT combines the Harris corner detection algorithm with SIFT, but their results could contain mismatches that are tackled by the RANSAC step. The RANSAC algorithm is customized for this problem by substituting the pure random sampling by sampling in separate grids and, also, by performing an efficient model validation strategy that can detect invalid models without checking all the input data. Experiments report that when compared with unmodified RANSAC, the average matching time decreases by 63.45%, the average matching accuracy increases by 15.66%, and the average deviation in pose detection decreases in all the coordinate axes.

The robotics subfield of unmanned aerial vehicles (UAVs) can also greatly benefit from advances in image matching. In [73], the authors propose a method that combines RANSAC with SURF [74] for the problem of matching images and test it on aerial images taken from UAVs. As in previously mentioned works, RANSAC is employed to refine the matches found by another method. In this case, SURF is the method used to detect features, and the authors find that it compares favorably to using SURF, SIFT, or ORB alone, although their experimental setup only includes a pair of aerial images. In [75], the authors present Prior Sampling and Sample Check RANSAC (PSSC-RANSAC), which incorporates prior knowledge of the sampling goodness coming from three different sources: texture magnitude, spatial consistency, and feature similarity. This prior sampling should possibly generate more correct samples. Furthermore, prior information on the collection of sample subsets is used to check them and rule out incompatible arrangements of subsets, yielding further improvements in speed. Their experiments on a dataset composed of images taken from online sources and collected by themselves show improvements over standard RANSAC and SVH-RANSAC [76]. Target tracking and following from a multirotor UAV is the subject of the research carried out in [77]. The paper presents an end-to-end architecture that combines: image acquisition to obtain the data to compute the transformation matrix, Recursive RANSAC to perform target tracking, a track selection process, and a controller for the target-following task. The physical setup is composed of a monocular camera, an inertial measurement unit, an altitude sensor, and an embedded computer, all of them into a multirotor UAV with a flight control unit. The system works under the assumption that the target is moving on a surface close to planar and with a velocity approximately constant. Their results in simulation suggest that the proposed pipeline is effective and robust to target modeling errors. Another use of the data collected by UAVs is the creation of digital surface models (DSM). In [78], the authors propose a RANSAC modification to improve image matching with a special interest in the quality of the photogrammetry needed to create digital surface models. They enhance RANSAC using an iterative least-squares-based loop, a similarity termination criterion, and a post-processing step. In the locally iterative least-squares-based loop, all inliers found in the previous iteration are used to recompute the model parameters. Then, a least-square solution to improve the model is applied, and the number of inliers is counted in each step until that number does not change. The loop stops when a predetermined maximum number of iterations is achieved, or if the inliers ratio is higher than a good enough threshold. This iterative least-squares-based loop improves the stability, convergence rate, and number of inliers of the found solution. Another termination criterion is defined for the RANSAC loop: if the similarity of the sets of inlier points between two consecutive RANSAC iterations is greater than 95%, the loop stops, saving running time. Finally, a post-processing step is performed to remove outliers in the final model. The authors find favorable comparison with RANSAC over a set of four aerial images taken by themselves.

The next research does not fall into the category of image matching, but it is also of interest to any application in need of data for training a model. Image augmentation is the process of generating images similar to those present in a dataset, through several transformations, often with the aim of providing more training data to machine learning algorithms [79]. In [80], the authors propose a hybrid RANSAC algorithm to create a mosaic from several single images. They take images from similar areas and perform feature matching using RANSAC, using the location of those features to blend the pictures to create new ones. The authors claim their method is well-suited for aerial photos and report an increase in image augmentation data compared with other techniques.

### 4.2. Shape Detection

One of the key capabilities needed for a robotic system able to interact in an intelligent way with its environment is the potential to detect and identify objects. This is in itself a vast field of research, fueled in the last years by the great interest of the big technological actors and the advent of deep learning. YOLO [81] represented a big leap for object detection in 2D images, and 3D versions have been proposed [82,83,84]. Other approaches based on 3D descriptors [85,86,87] or other deep learning architectures [88,89,90] have also been the subject of research. These approaches do not rely on a priori knowledge about the objects to be recognized, but in human-made objects, it is usual that familiar geometric shapes are prevalent. Even in nature, flat terrain or water reservoirs can be roughly characterized as planes.

An example of simple shape detection in nature can be found in [91], where the authors develop a method for monitoring the water level in a river for a flood warning system. A UAV records aerial images, and a dense point cloud is obtained from them using photogrammetric software. They fit the river surface plane using RANSAC, but to estimate the water level, a time-invariant reference in the scene with a known altitude is needed. They take a point in the road over a bridge with precise altitude information, but if this were not available, a recognizable feature in the scene should be used as a reference, at least to estimate water level change between point clouds taken at different times. Experiments are performed with data collected on ten separate dates over the course of a month, with different water levels. Testing different image resolutions, the authors find that low-resolution images provide a more detailed point cloud due to the fact that the alignment software detects more matching points. They speculate that this could be because detailed river flow and tree branch movements with the wind introduce undesirable noise in the images. A linear regression of the calculated water level against the reference water level shows $R^{2}=0.98$ for a slope of 0.95 and a standard deviation of 0.37 m. Another application in nature is the detection and delineation of trees presented in [92]. The LiDAR data are captured in an area of 1796 hectares during the flight of a Cessna. The models that they use to fit a tree are of a paraboloid, a cone, or another one that they call a shape-shifter, which is an interpolation between a cone and a paraboloid, performing filtering of local maxima before executing RANSAC. To compute the height of each tree, they use Hardy’s multiquadric method [93] to reconstruct the ground surface beneath the tree canopy. The authors report that their method, when applied to terrain with a mix of different tree species and is densely populated, yields tree counts similar to the inventory performed directly on the field. The difference is attributed mainly to small trees not detected by the LiDAR but that contribute less to the total counts.

Aerial imagery also has applications in urban areas in order to locate spaces of interest. One of the sources of renewable energy of great interest nowadays is solar energy. To find suitable places to place solar panels, in [94], the authors are interested in the analysis of the inclination and plane parameters of the roofs in an urban area. They apply RANSAC to data obtained from aerial photogrammetry and LIDAR data of three buildings taken by a UAV at a height of 80 m. The experiment shows that, while LIDAR data are less accurate than aerial photogrammetry, sometimes trees occlude parts of the roofs, circumstances in which LIDAR performs better than photogrammetry. The authors point out the importance of an accurate data source and find that irregular roof shapes are not detected correctly.

Another use case for urban areas is autonomous driving. If a vehicle is going to successfully navigate through a city, it is of paramount importance to correctly detect the traffic lanes. In [95], a real-time method for detecting all the lane markers in an image is presented. After filtering the image using selective oriented Gaussian filters, a RANSAC line fitting step provides initial guesses to another proposed fast RANSAC algorithm for fitting Bezier splines. A post-processing step to better localize and extend the spline is applied, with excellent results at a real-time rate of 50 Hz. In [96], the lane detection process starts with the application of inverse perspective mapping to change the camera perspective to a bird’s-eye view. This transforms the problem of detecting lanes into finding parallel lines separated by a fixed and given distance. Candidate lanes are found applying the Hough transform, and the results are further refined with RANSAC. A Kalman filter helps to remove minor perturbations. Experiments on the streets and highways around Atlanta in various traffic conditions show that the approach achieves good performance. In [97], the authors introduce ridgeness, a low-level image descriptor, which assigns high values along the center lines of the lane markings and low values close to the boundaries in the longitudinal direction. Then, RANSAC is applied using ridgeness and orientation as input to find the hyperbolas which correspond to the projection of the actual lane markings. They claim good results under different driving circumstances and straight and curved lanes. In [98], the model is refined to detect left and right lanes simultaneously, and extra information is returned: lane width, lane curvature, vehicle yaw angle, and lateral offset with respect to the lane medial axis. In [99], the images obtained by the car camera are split into two areas: a far-field area and a near-field area. In the near-field area, the lanes are detected by the Hough transform for lines, while in the far-field area, the lanes are observed as curves and are therefore detected by RANSAC using a hyperbolic model. Experiments under different driving conditions yield good results. A survey of advances in vision-based lane detection, covering works in which RANSAC was employed, is presented in [100].

Cable inspection is one of the tasks that autonomous underwater vehicles must perform. In [101], Crossline Correction Nonlinear RANSAC (CCNL-RANSAC) is presented to tackle the problem of detecting objects with a shape similar to a curved line. As underwater imagery often suffers from blurring, low contrast, nonuniform illumination, and noise, their approach performs a preprocessing step in order to improve the quality of the acquired images. Afterward, an adaptive edge detector based on Canny [102] and Otsu’s method [103] is run, and then CCNL-RANSAC is applied. CCNL-RANSAC integrates a preliminary inlier estimation module with a nonlinear fitting model and a final crossline correction procedure to remove false positives that could arise. Experiments with images collected in a boat tank at a university facility show that the algorithm can detect underwater curved-line objects with a success rate of 95% to a distance of 21 m.

Plane detection is one of the most usual applications of RANSAC, which is also of interest for autonomous driving or any navigation in urbanized terrain. When fitting several planes from point cloud data, it is possible that sometimes a spurious plane that shares inliers from other legitimate planes is erroneously detected. This is a usual fact when detecting curbs or ramps in urban scenery. CC-RANSAC [104] addresses this issue by changing the way that the fitness of a candidate plane is computed: instead of counting the total number of inliers, it only considers those that lie in the largest connected components. CC-RANSAC fails if two areas of the scene corresponding to planar surfaces are too close to each other, because the connected components of the two areas could join together. NCC-RANSAC [105] overcomes some of the limitations of CC-RANSAC, performing a check of the normal vectors in the area to find if they are coherent with the fitted plane. After obtaining a collection of candidate planes, a recursive clustering process is performed to grow each one of the candidates. The authors validate the robustness of their approach with a probabilistic model and obtain a very high rate of success. In indoor or outdoor mobile robotics, ground detection is a common task. In [106], the authors take advantage of the fact that all the other objects in the scene are always above the ground to define an asymmetric kernel as the score function for RANSAC. The ground parameter is estimated by maximum likelihood estimation, where the log-likelihood is modeled as an asymmetric Gaussian kernel. Experiments show that the proposed model is fast as well as robust.

A general method aimed to improve the plane segmentation process in point clouds is described in [107]. After downsampling the point cloud using the voxel grid method, the authors estimate the normal at each point and refine such estimate employing the Mean Shift algorithm [108]. After that, RANSAC, with the constraints given by the normals, is applied to find the plane. Experiments with data acquired by the authors show good results and practical value in industrial settings. In [109], the authors present a Python library for segmenting assets in an industrial indoor scene. They use RANSAC for plane segmentation and then employ parallelism and perpendicularity between the detected planes, along with the sensor orientation, to find the ground, ceiling, and walls of a room. Other elements in the scene can also be detected by analyzing further relationships. In [110], pairwise orthogonal planes are defined as a primitive shape and then detected directly by RANSAC. The parameters of the shape are a point and two unit orthogonal normals, and they formulate the problem of refining each candidate model as a nonlinear least-square optimization task, which is solved by employing the Levenberg–Marquardt algorithm [111]. The candidate models are generated by RANSAC. Experiments on Stanford 3D large-scale dataset [112] show that the method is efficient, even for extracting small planes. Moreover, the authors claim that their approach can also be adapted to deal with other geometry structures.

Building Information Modeling (BIM) is a process that involves the generation and management of digital information about the physical and functional characteristics of buildings. As that information is not directly available from structures in which BIM was not present in the design and building process, it is important to generate BIMs from existing places in an efficient manner [113]. In [114], the authors present a method to apply RANSAC iteratively, where each iteration takes as input the inlier set of the previous one, to automatically extract the height and the layout of a room. They report promising results in a cloud extracted from the ISPRS dataset [115], although they point out that a limitation of their model is that it is limited to rooms with polygonal layouts and flat surfaces.

### 4.3. Hardware Acceleration

Making software run faster is one of the main goals driving the new technology industry. From the point of view of pure software engineering research into algorithmic theory, computational gains could be achieved that are to some extent independent of the underlying hardware. However, advances in hardware also make it possible to leverage the new capabilities of modern processors and dedicated processing units to achieve running speeds orders of magnitude above a single CPU. It is possible to write parallel software somehow independent of the hardware, but this approach has been gradually superseded by the advent of GPUs and TPUs: dedicated graphical processors and tensor processors, respectively [116]. GPUs were designed for the acceleration of graphics rendering computations but now have a prominent place in artificial intelligence research and applications. TPUs have been specifically designed for tensor operations in deep learning. FPGAs [117] are programmable hardware that could very efficiently perform a specific task and are suitable for embedded devices.

Several steps of the RANSAC procedure are very suitable for parallelization. For example, the generation of hypotheses or the scoring of those hypotheses against the input data. We suspect that some straightforward implementations do not have enough relevance for publication as a research article, and that is the reason for the lack of description of such obvious variations in the literature. However, some articles deserve a mention in this section.

In [118], the authors directly implement RANSAC in hardware using Verilog, a hardware description language (HDL) that is used to model electronic systems. Their design implements random sampling by using the multiple-input signature register (MISR) and the index register. At the same time, the matrix triangularization operation needed by the forward elimination is implemented by a systolic array [119], which is a piece of hardware specifically built for fast and efficient implementations of regular algorithms that perform the same task with different data at different timestamps. The authors report speeds, in simulated hardware, of 30 frames (1024 × 1024 pixels) per second for computing the homography between pairs of images.

RANSAC has been implemented in FPGAs. In [120], an implementation for real-time affine geometry estimation is introduced. The main task chosen to be accelerated was the fitness scoring function, where the authors claim that the speed-up factor increases with input data size. Another layer of acceleration was implemented over the iterations of the full RANSAC workflow, therefore increasing the probability of obtaining good estimation results in the same running time. Experiments with video frames extracted from the Unmanned Aerial Vehicle Database [121] show increases in the speed of about 11.4 times for 100 data points, the system being able to handle a video stream of 30 fps. In [122], another FPGA implementation for the same problem is described, with their architecture able to reject false correspondences between similar images. Three modules are defined: transformation matrix calculator, inliers count calculator, and RANSAC controller. The transformation matrix calculator computes the affine transformation parameters from three samples from the set of feature matches, the inliers count calculator computes the number of inliers for the current transformation matrix, and the RANSAC controller reads samples from the array of initial matches and stores them in the array of random samples. The execution of RANSAC takes a number of clock cycles equal to the number of selected random samples. The authors report a running time of less than 23 ms for the processing of 128 initial matches, with a supported video streaming rate of at least 43 fps. Their architecture has been tested in simulation and on hardware (Altera Cyclone IV). These same authors later present another FPGA implementation for real-time SIFT [123] matching and RANSAC to improve over the previous solutions to the problem of identifying the correct correspondences between feature points between consecutive video frames [124]. The feature descriptors from each frame are stored, and when a new feature is extracted from the next frame, its descriptor is compared with those corresponding to the previous frame. If the matching criterion is fulfilled, then the coordinates of the match are stored in on-chip RAM. A moving window of size 16 is defined in the shift register structure to store and shift the feature descriptors. This facilitates the fit in the processing pipeline of the matching procedure, by supporting a standard number of parallel comparisons between features. Several sets of moving windows are defined concurrently, allowing for an effective size of 128. Using Altera Cyclone IV again, they achieve a processing rate of 40 fps for VGA resolution (640 × 480).

It is also possible to parallelize RANSAC using APIs that could access to the parallel capabilities of modern processors or GPUs. In [125], the task of fitting a plane in a 3D point cloud is implemented in three different paradigms: OpenMP, POSIX threads, and CUDA. Their goal is to analyze the relative performance of these three approaches over a collection of point clouds collected by the same authors in an indoor environment. The point clouds cover different spaces: living room, kitchen, hallway, saloon, room, and furniture. In addition to three usual metrics in evaluating search strategies (Precision, Recall, and F-Score), they also report other two standard metrics in parallelism (Runtime and Speedup). In their study, they find that CUDA over NVidia GPUs is the best option, with good results in all the metrics. POSIX threads are a better option than OpenMP if the researcher is willing to program to a low level to profit from the fine control that OpenMP does not allow for, it being too high-level.

## 5. Software

Researchers working on RANSAC variants have, sometimes, made their code available to the community independently, giving rise to a fragmented ecosystem with implementations in several languages that often lack maintenance. At the same time, popular computer vision libraries have implemented RANSAC variants for shape detection or image matching. In this section, we present available software that could be of interest to the robotics practitioner willing to test RANSAC capabilities.

### 5.1. OpenCV

OpenCV is a library of functions aimed to tackle common tasks in computer vision, especially focused on real-time performance. The project was started by Intel, and it is now released under the open-source Apache 2 license. It is cross-platform and offers C++ and Python APIs. Some operations support GPU accelerations. Robot Operating System (ROS) (http://wiki.ros.org/ accessed on 23 November 2022) provides an easy way to integrate OpenCV calls in ROS developments (http://wiki.ros.org/vision_opencv accessed on 23 November 2022).

The documentation is not very exhaustive, but the *calib3d* module, which is in charge of finding the transformation matrix between different points of view, provides several RANSAC-related implementations (https://docs.opencv.org/4.x/d1/df1/md__build_master-contrib_docs-lin64_opencv_doc_tutorials_calib3d_usac.html accessed on 23 November 2022).

Choices for the sampling method in the general RANSAC procedure include the standard sampling of RANSAC, or the alternatives of PROSAC, NAPSAC, or progressive-NAPSAC [126]. The score method could also be set to the standard of RANSAC, or those of MSAC, MAGSAC or the least median of squared error distances. A local optimization step using LO-RANSAC, Graph-Cut RANSAC, or the sigma consensus of MAGSAC++ is available to the user. Finally, sequential probability ratio test (SPRT) verification evaluates a model on randomly drawn points using statistical properties obtained from the probability of a point being inlier, the average number of output models, etc. This could speed up the process in a significant manner because a bad model could be rejected without computing the error for every point.

### 5.2. Point Cloud Library

The Point Cloud Library (PCL) is an open project for 2D and 3D image and point cloud processing [127]. It is released under the three-clause BSD license, which permits research and commercial use free of any fees. The authors claim to have implemented state-of-the-art algorithms in the areas of registration, feature estimation, filtering, surface reconstruction, segmentation, and model fitting. Some examples of their capabilities that could be of interest to the robotic community are outlier filtering from noisy point clouds, scene segmentation, and geometric descriptors computation. It is written in C++ and has been compiled on Linux, macOS, Windows, and Android. As with OpenCV, ROS permits integration of PCL in ROS-based software (http://wiki.ros.org/pcl/ accessed on 23 November 2022).

PCL implements several sample consensus methods applied to different models (https://pointclouds.org/documentation/group__sample__consensus.html accessed on 23 November 2022). It is possible to call the different methods by their corresponding individual implementation or to create a segmentation of objects and pass the method and model types as parameters. In Table 2, we show all the available methods along with the name they receive according to the PCL API. The geometric models, along with the number of coefficients needed to describe them, are shown in Table 3.

The four coefficients of the plane model must be provided in Hessian normal form: $n_{x}$, $n_{y}$, $n_{z}$, and *d*, where $n_{x}$, $n_{y}$, and $n_{z}$ are the components of the unit normal vector, and *d* is the signed distance from the plane to the origin. The sign of *d* determines the side of the plane on which the origin is located. If p>0, the origin is in the half-space determined by the direction of the normal, and if p<0, it is in the other half-space. The six coefficients of the line model are given by a point on the line (*p*) and the direction of the line (*d*) as [$p_{x}$, $p_{y}$, $p_{z}$, $d_{x}$, $d_{y}$, $d_{z}$]. The 2D circle’s three coefficients are given by its center (*c*) and radius (*r*) as: [$c_{x}$, $c_{y}$, *r*], while the seven coefficients of the 3D circle are given by its center (*c*), radius (*r*), and normal (*n*) as [$c_{x}$, $c_{y}$, $c_{z}$, *r*, $n_{x}$, $n_{y}$, $n_{z}$]. The four coefficients of the sphere are given by its 3D center (*c*) and radius (*r*) as: [$c_{x}$, $c_{y}$, $c_{z}$, *r*]. In the case of the cylinder model, the seven coefficients must be given by a point on its axis (*p*), the axis direction (*d*), and a radius (*r*), as [$p_{x}$, $p_{y}$, $p_{z}$, $d_{x}$, $d_{y}$, $d_{z}$, *r*], while for a cone model, its seven coefficients correspond to a point of its apex (*a*), the axis direction (*d*), and the opening angle (*o*), as: [$a_{x}$, $a_{y}$, $a_{z}$, $d_{x}$, $d_{y}$, $d_{z}$, *o*]. For parallel lines and parallel and perpendicular planes, the extra constraints, in addition to the model coefficients, are the axis with respect to being parallel or perpendicular and the maximum angular deviation tolerated. For a normal plane, the surface normals at each tentative inlier point are computed and have to be parallel to the normal of the tentative plane, within a maximum specified angular deviation. The normal sphere model adds additional surface normal constraints. The normal parallel plane restricts the normal plane, with the constraint that such a normal plane has to be parallel to a given axis. There are plans to implement a torus model as well as parallel lines.

The PCL maintainers do not plan (as of version 1.12.1) to provide widespread GPU support due to the difficult integration of NVidia libraries with their CI/CD practices.

### 5.3. Other Software

Some of the authors of the variants or applications mentioned so far have provided a software implementation of their algorithms. In Table 4, there is a list of available implementations. It is worth noticing that GraphCut-RANSAC has also been implemented in OpenCV.

Open3D [128] aims to be an alternative to PCL, focusing on making its use easy and also providing the capability of rapid prototyping. It implements a plane segmentation function that relies on RANSAC, although it is not very sophisticated in the current version (0.16), not permitting other geometric models apart from the plane. Open3D is the library behind indoor3D, the library for processing 3D data from indoor scenes [109]. Another Python library not mentioned so far is pyRANSAC-3D [129], employed, for example, in [91]. The characteristics of these libraries, along with those previously presented, are summarized in Table 5.

## 6. Discussion and Conclusions

Methods from the RANSAC family have been widely studied and applied to problems arising in robotic applications. The interested researcher could easily implement a simple model with open-source software, but even programming a RANSAC variant from scratch should not be such a daunting task as with other models, as they are comparatively simpler than, for example, deep learning approaches.

In the literature in general, and in the open source tools in particular, we miss more general support of parallel implementations. In the deep learning era, GPU acceleration is widespread, and RANSAC could benefit greatly from these techniques, as the method iterations or the model score function could be parallelized rather easily.

Plane fitting is the most common shape detection task, likely due to the simplicity of the geometric model and its ubiquity in human-made structures. While detectors of other simple geometric shapes, such as spheres, cones, or cylinders have also been implemented in open-source software, the field could also benefit from research into modeling more complex shapes.

Other machine learning methods such as deep neural networks are nowadays used in all kinds of areas due to their unquestionable performance, but RANSAC is conceptually simpler and easier to implement when the model to recognize is known in advance and suitable for parameterization. For example, if a robotics application needs to recognize objects with no shape restriction, deep neural networks would be the default choice, while if those shapes are known in advance and simple enough to parameterize, RANSAC could be a good option. RANSAC could also be used as a preprocessing step for filtering the floor, ceiling, or walls of a point cloud taken indoors, before deep learning takes charge of segmenting the remaining data.

In contrast with the trial-and-error approach of hyperparameter tuning of deep neural networks, theoretical results have been achieved of RANSAC parameters depending on the a priori knowledge of the data distribution. For example, the number of iterations needed for a given probability of randomly drawing all inlier points in some iteration can be computed if the ratio of inlier points is known in advance.

In short, the main conclusions could be summarized as:

1. RANSAC is a good alternative to deep learning approaches when the model whose parameters we want to estimate is known in advance, which is the case, e.g., of shape matching of simple objects in many robotic applications.
2. Theoretical analysis of the probability of estimating the model parameters is possible, and this can lead to optimal use of resources in embedded devices or real-time applications.
3. Open-source implementations of RANSAC variants are available for the robotics community.
4. Research in parallelization and hybrid approaches with deep learning methods could be promising.

## Figures and Tables

**Table 1:** RANSAC variants grouped by the metric they aim to improve.

Focus on	RANSAC Variant
Accuracy	MSAC (M-estimator SAC) [15]
	MLESAC (Maximum Likelihood SAC) [16]
	MAPSAC (Maximum A Posterior Estimation SAC) [17]
	LO-RANSAC (Locally Optimized RANSAC) [18]
	QDEGSAC (RANSAC for Quasi-degenerate Data) [19]
	Graph-Cut RANSAC [20]
Speed	NAPSAC (N Adjacent Points SAmple Consensus) [21]
	Randomized RANSAC with $T_{d,d}$ test [22]
	Guided-MLESAC [23]
	RANSAC with bail-out test [24]
	Randomized RANSAC with Sequential Probability Ratio Test [25]
	PROSAC (Progressive Sample Consensus) [26]
	GASAC (Genetic Algorithm SAC) [27]
	1-point RANSAC [28]
	GCSAC (Geometrical Constraint SAmple Consensus) [29]
	Latent RANSAC [13]
Robustness	AMLESAC [30]
	u-MLESAC [31]
	Recursive RANSAC [32]
	SC-RANSAC (Spatial Consistency RANSAC) [33]
	NG-RANSAC (Neural-Guided RANSAC) [34]
	LP-RANSAC (Locality-preserving RANSAC) [35]
Optimality	Optimal Randomized RANSAC [36]
	Optimal RANSAC [37]

**Table 2:** List of sample consensus methods available in PCL (as of 1.12.1 version).

API Name	Method
SAC_RANSAC	RANdom SAmple Consensus
SAC_LMEDS	Least Median of Squares
SAC_MSAC	M-Estimator SAmple Consensus
SAC_RRANSAC	Randomized RANSAC
SAC_MLESAC	Maximum LikeLihood Estimation SAmple Consensus
SAC_PROSAC	PROgressive SAmple Consensus

**Table 3:** List of models available in PCL (as of 1.12.1 version).

API Name	Model	Coefficients	Constraints
SACMODEL_PLANE	Plane	4	No
SACMODEL_LINE	Line	6	No
SACMODEL_CIRCLE2D	Circle	3	No
SACMODEL_CIRCLE3D	Circle	7	No
SACMODEL_SPHERE	Sphere	4	No
SACMODEL_CYLINDER	Cylinder	7	No
SACMODEL_CONE	Cone	7	No
SACMODEL_PARALLEL_LINE	Line	6	Yes
SACMODEL_PERPENDICULAR_PLANE	Plane	4	Yes
SACMODEL_NORMAL_PLANE	Plane	4	Yes
SACMODEL_NORMAL_SPHERE	Sphere	4	Yes
SACMODEL_PARALLEL_PLANE	Plane	4	Yes
SACMODEL_NORMAL_PARALLEL_PLANE	Plane	4	Yes
SACMODEL_STICK	Line	6	Yes

**Table 4:** Software implementations of RANSAC variants.

RANSAC Variant	Language	Code
GraphCut-RANSAC [44]	C++	https://github.com/danini/graph-cut-ransac accessed on 23 November 2022
GCSAC [29]	C++	http://mica.edu.vn/perso/Le-Van-Hung/GCSAC/index.html accessed on 23 November 2022
Latent RANSAC [13]	C++	https://github.com/rlit/LatentRANSAC accessed on 23 November 2022
Optimal RANSAC [37]	Matlab	https://www.cb.uu.se/~aht/code.html accessed on 23 November 2022
RANSAC-Flow [65]	Python (PyTorch)	https://github.com/XiSHEN0220/RANSAC-Flow accessed on 23 November 2022

**Table 5:** Libraries with RANSAC APIs.

Library	Language	URL
OpenCV [130]	C++, Python	https://opencv.org/ accessed on 23 November 2022
PCL [127]	C++	https://pointclouds.org/ accessed on 23 November 2022
Open3D [128]	C++, Python	http://www.open3d.org/ accessed on 23 November 2022
pyRANSAC-3D [129]	Python	https://github.com/leomariga/pyRANSAC-3D/ accessed on 23 November 2022
indoor3D [109]	Python	https://github.com/rsait/indoor3d accessed on 23 November 2022

## Data Availability

Not applicable.
